# *Selaginella tamariscina* Ethanol Extract Attenuates Influenza A Virus Infection by Inhibiting Hemagglutinin and Neuraminidase

**DOI:** 10.3390/nu16142377

**Published:** 2024-07-22

**Authors:** Won-Kyung Cho, Hee-Jeong Choi, Jin Yeul Ma

**Affiliations:** Korean Medicine (KM) Application Center, Korea Institute of Oriental Medicine, 70 Chemdanro, Dong-gu, Daegu 41062, Republic of Korea; chj1901@kiom.re.kr

**Keywords:** *Selaginella tamariscina*, influenza A virus, cytopathic effect, hemagglutinin, neuraminidase

## Abstract

*Selaginella tamariscina* is a perennial plant that is used for diverse diseases. This study investigated whether *Selaginella tamariscina* has an antiviral effect against influenza A virus (IAV) infection. We used green fluorescent protein (GFP)-tagged influenza A virus (IAV) to examine the effect of *Selaginella tamariscina* ethanol extract (STE) on influenza viral infection. Fluorescence microscopy and flow cytometry showed that STE potently represses GFP expression by the virus, dose-dependently. STE significantly inhibited the expression of the IAV M2, NP, HA, NA, NS1, and PB2 proteins. Time-of-addition and hemagglutination inhibition assays showed that STE has an inhibitory effect on hemagglutinin and viral binding on the cells at an early infection time. In addition, STE exerted a suppressive effect on the neuraminidase activity of the H1N1 and H3N2 IAVs. Furthermore, dose-dependently, STE inhibited the cytopathic effect induced by H3N2, as well as by H1N1 IAV. Especially in the presence of 200 µg/mL STE, the cytopathic effect was completely blocked. Our findings suggest that STE has antiviral efficacy against IAV infection; thus, it could be developed as a natural IAV inhibitor.

## 1. Introduction

Influenza viruses are responsible for respiratory diseases that cause symptoms such as coughing, fever, sore throat, chills, muscle pain, and fatigue. Millions of people become infected with the influenza viruses, resulting in up to 650,000 deaths in the world every year [1]. Influenza viruses have segmented negative single-strand RNA and belong to the Orthomyxoviridae family [2]. Influenza A virus (IAV) is the major causing agent of human flu and is classified into subtypes based on hemagglutinin (HA) and neuraminidase (NA) [2,3]. The segmented genome of IAV frequently produces gene reassortment events such as antigenic drift and shift during viral RNA gene expression [4]. Antigenic drift and shift in the HA and NA of IAV make new types of viruses unpredictable each year [5]. NA inhibitors, such as zanamivir, oseltamivir, and peramivir, and PA inhibitors, such as baloxavir, are used to treat IAV infections. Still, the variants caused by mutations in viral RNA replication or resistance to NA inhibitors pose a challenge in the treatment of IAV-related diseases [6,7,8]. To date, many types of natural herbs have been reported to have the potential to prevent IAV infection by targeting IAV proteins, including NA, NS1, HA, NP, PA, and PB [9,10,11,12,13]. However, a perfect natural antiviral agent that can overcome the shortcomings of NA inhibitors has still not been developed.

*Selaginella tamariscina* (ST), an evergreen perennial plant widely grown in East Asia, has long been utilized to treat various diseases, including hemoptysis in pulmonary disease, traumatic and gastrointestinal bleeding, leukorrhea, and cancer [14]. ST is currently used as a component of traditional Chinese medicine to treat rhinitis/sinusitis, arthritis, and hemorrhoids in China [14]. In addition to medicine, ST is used as an ingredient in cosmetics, food, and tea. Several studies have reported that ST has antiviral effects against the respiratory syncytial virus [15], hepatitis B [16], and coxsackie viruses [17]. However, the inhibitory effect of ST on IAV has not been investigated so far. Here, we report for the first time that *Selaginella tamariscina* ethanol extract directly inhibits influenza A virus infection, by not only interfering with hemagglutinin but also by inhibiting neuraminidase activities.

## 2. Materials and Methods

### 2.1. Herbal Extract Preparation and HPLC Analysis

The preparation of *Selaginella tamariscina* ethanol extract (STE) and the HPLC analysis to verify STE were described in a previous report [18] by our research group. Briefly, dried ST was obtained from YeongCheon Hyundai Herbal Market (Yeongcheon, Republic of Korea) and verified by Professor Ki Hwan Bae of the College of Pharmacy, Chungnam National University (Daejeon, Republic of Korea). An amount of 50 g of ST was extracted by shaking in 70% ethanol at 40 °C for 24 h. After filtration with 150 μm testing sieves (Retsch, Haan, Germany), the solution was freeze-dried to evaporate the ethanol solution and stored at −20 °C. For antiviral experiments, STE was dissolved at 100 mg/mL in 50% DMSO. To identify the main compound in STE, an HPLC analysis was conducted as described in the previous report [18]. Amentoflavone was detected as a major compound at 43.00 min in an HPLC chromatogram. The content of amentoflavone was 4.65%.

### 2.2. Cell Culture and Influenza Viruses

The RPMI (Le Roswell Park Memorial Institute medium, Hyclone, Logan, UT, USA), containing fetal bovine serum (10%) and penicillin and streptomycin (100 U/mL), was used for the culture of RAW 264.7 cells (ATCC TIB-71). The influenza A/PR8/34 (H1N1) and green fluorescent protein (GFP)-expressing influenza A/PR8/34 viruses were kindly provided by Professor Jong-Soo Lee (Chungnam National University, Daejeon, Republic of Korea), and the HBPV-VR-32 (H3N2) influenza virus was obtained from the Korea Bank for Pathogenic Viruses (KBPV). The viruses were propagated using the allantoic fluid of a 10-day-old chicken embryo.

### 2.3. Cytotoxicity Assays

CCK-8 assay was used to check the toxicity of STE on the cells according to the manufacturer’s instructions (Dojindo, Rockville, MD, USA). RAW 264.7 cells grown in a 96-well plate (1 × 10^5^ cells/well) were treated with STE (1 to 1000 µg/mL) and incubated for 24 h at 37 °C. After the addition of 10 μL of the CCK-8 reagent to the cells for 2 h, the absorbance at 450 nm was determined by a spectrophotometer (Promega, Madison, WI, USA). 

### 2.4. Anti-Influenza Viral Assay 

The PR8-GFP, H1N1, or H3N2 IAVs were mixed with STE for 1 h at 4 °C. The mixtures were added to the cells for 2 h at 37 °C and the cells were washed with PBS to remove the remaining virus. The cells were further incubated until the formation of the cytopathic effect (CPE) or the expression of GFP. GFP expression was evaluated using FACS analysis, and CPE reduction was examined using a CCK-8 assay.

### 2.5. Immunofluorescence Staining

The RAW 264.7 cells, cotreated with a mixture of STE (100 µg/mL) and PR8-GFP IAV (10 MOI), were fixed with methanol for 10 min and 4% paraformaldehyde at room temperature. The cells were washed three times with PBS and blocked with PBS containing 1% BSA for 30 min. The cells were incubated with primary antibodies specific for IAV proteins, including M2, NP, NS1, HA, NA, and PB2 (GeneTex, Irvine, CA, USA), overnight at 4 °C. After washing with 0.05% Tween 20-containing PBS (PBST), the cells were incubated with an Alexa Fluor 594-conjugated secondary antibody (Thermo Fisher Scientific, Waltham, MA, USA) for 1 h in the dark. The cells were further incubated with Hoechst 33342 for 5 min in the dark. The images of the red color of the IAV proteins and the blue color of the nuclei were obtained by fluorescence microscopy. 

### 2.6. Flow Cytometry

The cells coinfected with PR8-GFP IAV and STE were collected and washed three times with PBS. The cells fixed in 4% paraformaldehyde were analyzed by a CytoPLEX flow cell counter (Beckman Coulter Inc., Pasadena, CA, USA). 

### 2.7. Time-of-Addition Assay

A time-of-addition assay was conducted as described in a previous study [19]. Briefly, for the virus attachment step, RAW 264.7 cells in 12 wells (5 × 10^5^ cells/well) were coincubated with 100 µg/mL STE and 10 MOI PR8-GFP IAV for 30 min at 4 °C. After removing the mixture with PBS washing, the cells were incubated for 24 h at 37 °C. For the virus entry step, STE was added to the cells, which were preincubated with PR8-GFP IAV for 30 min at 4 °C. After further incubation for 30 min at 37 °C, the cells were washed with PBS and further incubated for 24 h at 37 °C. For the virucidal effect, STE, PR8-GFP IAV and STE were preincubated for 30 min at 4 °C, and the cells were coinfected with the mixtures for 30 min at 37 °C. After washing with PBS, the cells were further incubated for 24 h at 37 °C. The images of the cells were captured by fluorescence microscopy (200× magnification). The expression of GFP was analyzed using flow cytometry with paraformaldehyde-fixed cells.

### 2.8. Hemagglutination (HA) Assay

The H1N1 IAV (10 MOI) and STE were mixed for 1 h at 4 °C. The cells were cotreated with the mixtures and incubated for 2 h at 37 °C. After the removal of the remaining virus and STE with PBS washing, the cells were incubated for 24 h at 37 °C. Serially diluted supernatant and 1% chicken RBCs (Innovative Research, Inc., Southfield, MI, USA) were mixed and incubated in a round-bottomed 96-well plate for 1 h at room temperature. The images of the plates with red blood cell agglutination were obtained photographically.

### 2.9. Neuraminidase (NA) Inhibition Assay

The neuraminidase (NA) inhibition assay was performed using the NA-Fluor influenza Neuraminidase Assay Kit (Life Technologies, Carlsbad, CA, USA). Briefly, STE or oseltamivir carboxylate (as a positive control), which was serially diluted with buffer, was added to a 96-well black plate. The H1N1 or H3N2 IAV was added to each well, mixed, and incubated for 30 min at 37 °C. Each sample was incubated with NA-Fluor substrate for 1 h at 37 °C and terminated using the stop solution. NA activity was measured using a fluorescence spectrometer (Promega, Madison, WI, USA), with an excitation of 365 nm and an emission of 445 nm.

## 3. Results

### 3.1. STE Inhibits Influenza A Viral Infection in a Dose-Dependent Manner

**Figure 1:**
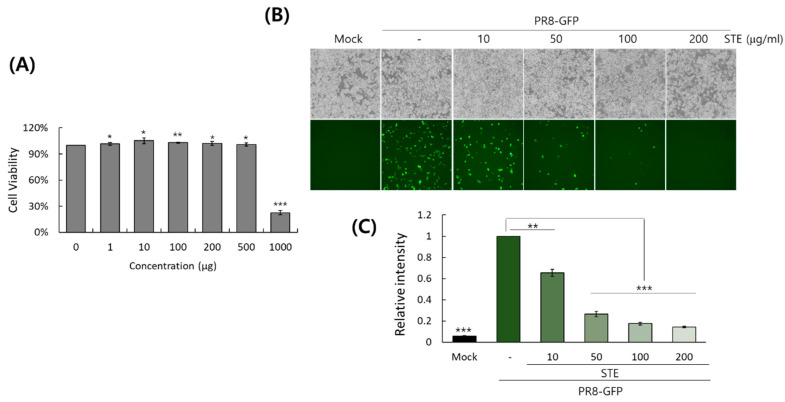
Cytotoxicity and antiviral effect of *Selaginella tamariscina* ethanol extract (STE) in RAW 264.7 cells. (**A**) The toxicity of STE in the cells was evaluated using a CCK-8 assay. The data represent the mean ± SD from three independent experiments. The unpaired Student *t*-test was used to assess the statistical significance. * *P* < 0.5, ** *P* < 0.05, and *** *P* < 0.005, compared with the untreated control. (**B**,**C**) The cells were cotreated with PR8-GFP IAV and STE. The effect of STE on PR8-GFP IAV infection was evaluated by comparing GFP expression using a fluorescence microscope (**B**) and FACS analysis (**C**). The data represent the mean ± SD from three independent experiments. The unpaired Student *t*-test was used to assess the statistical significance. ** *P* < 0.005, and *** *P* < 0.0005, compared with the virus-infected control.

Before investigating the effect of STE on influenza virus infection, we examined the toxicity on the RAW 264.7 cells. As presented in Figure 1A, STE was found to be nontoxic up to 500 µg/mL in the cells; therefore, we explored the effect of STE on IAV infection up to 200 µg/mL. The anti-viral effect of STE was examined in RAW 264.7 cells using the GFP-tagged influenza A virus (PR8-GFP IAV). Most studies use MDCK or A549 cells for influenza virus infection experiments. However, in this study, we used RAW 264.7 cells because they are also readily infected with IAV and their small cell size facilitates relative comparisons in dose-dependent experiments. As shown in Figure 1B, PR8-GFP IAV expressed high levels of GFP in the absence of STE. However, GFP expression was significantly reduced by STE, in a concentration-dependent manner. The effect of STE on PR8-GFP IAV infection was confirmed by FACS analysis. Figure 1C shows that STE significantly reduces GFP expression by viral infection, consistent with Figure 1B. STE showed a 50% effective concentration (EC_50_) of 14.5 ± 2.0 µg/mL on GFP expression by viral infection. We also confirmed dose-dependent the anti-influenza viral effect of STE in MDCK and A549 cells after checking the cytotoxicity on them (Figures S1 and S2). These results suggest that STE has a potent anti-viral effect against PR8-GFP IAV infection. 

### 3.2. Inhibitory Effect of STE on Influenza H1N1 and H3N2 Viruses

**Figure 2:**
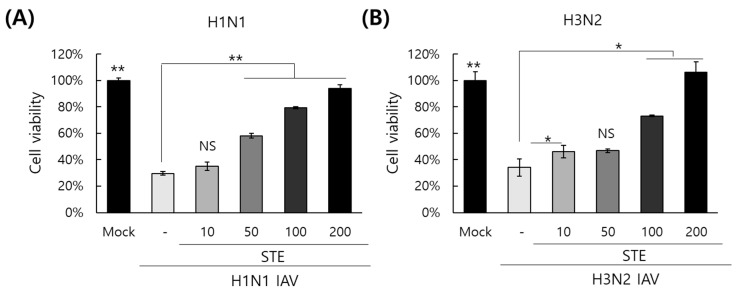
STE protects the cells from the cytopathic effect induced by H1N1 (**A**) and H3N2 (**B**) influenza virus infection. STE at the indicated concentrations or medium (mock) was mixed with H1N1 or H3N2 IAV before infection of the cells. The cells infected with the mixture were incubated until the cytopathic effect formed. The cell viability was determined via a CCK-8 assay. The data represent the mean ± SD from three independent experiments. The unpaired Student *t*-test was used to assess the statistical significance. * *P* < 0.05, ** *P* < 0.005; NS, no significance.

To investigate whether STE could inhibit other types of influenza viral infection, we checked the effect of STE on wild-type IAV infection by comparing the cytopathic effect. In the absence of STE, the H1N1 and H3N2 IAVs induced cytopathic effects in the cells (Figure 2). The viability of cells infected with H1N1 or H3N2 IAV was reduced to 30%, compared to the uninfected cells. However, in the presence of STE, the cell viability was significantly increased through the blockage of the cytopathic effect by viral replication, dose-dependently. Especially at a concentration of 200 µg/mL of STE, the cell viabilities in the cells infected with the H1N1 or H3N2 IAV were recovered up to 100%, compared to the uninfected cells. These results indicate that STE has a potent inhibitory effect against both H1N1 and H3N2 influenza virus infections.

### 3.3. STE Represses Influenza Viral Protein Expression

**Figure 3:**
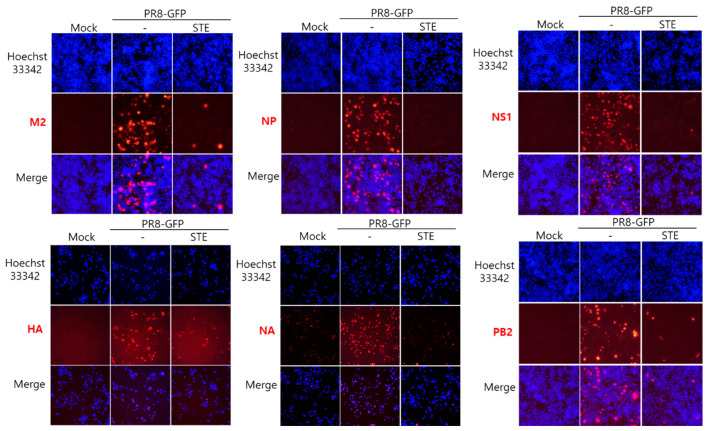
STE suppresses IAV protein expression. STE and PR8-GFP IAV were incubated for 1 h at 4 °C. The cells were coinfected with the mixture for 24 h at 37 °C. The cells were fixed and detected with antibodies against IAV proteins, including M2, NP, NS1, NA, HA, and PB2 (red color). To detect the nuclei, the cells were stained with Hoechst 33342 (blue color). The co-localization images of red viral proteins and blue nuclei were captured using a fluorescence microscope.

Next, we explored the effect of STE on IAV protein expression using the cells infected with PR8-GFP IAV, with or without STE. Immunofluorescence analysis with antibodies specific for IAV proteins showed that STE significantly decreased IAV proteins, including M2, NP, NS1, HA, NA, and PB2 (Figure 3). This result suggests that STE strongly represses influenza A viral protein expression. 

### 3.4. Effect of STE on Viral Attachment, Entry, or Virucidal Stages

**Figure 4:**
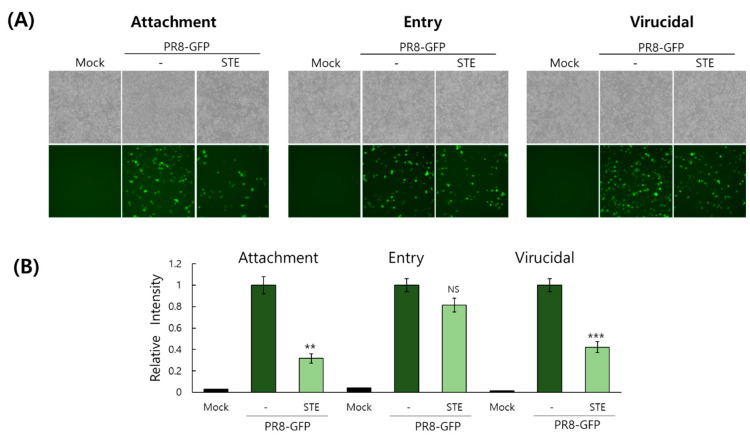
STE affects the virus attachment and entry and directly kills the virus at an early phase. The cells were cotreated with STE (100 µg/mL) and PR8-GFP IAV (10 MOI) to examine the effect of STE on viral attachment, entry, or virucidal stage. The detailed time-of-addition methods were described in the Materials and Methods Section. The images of GFP-expressing cells were obtained using brightfield and fluorescence microscopy (**A**). The cells fixed with paraformaldehyde were analyzed by flow cytometry (**B**). The data represent the mean ± SD from three independent experiments. The unpaired Student *t*-test was used to assess the statistical significance. ** *P* < 0.005, *** *P* < 0.0005; NS, no significance.

Because STE exhibited a potent inhibitory effect against IAV infection in the cotreatment condition, we conducted a time-of-addition experiment to elucidate whether STE could affect viral binding and entry on the cells at an early infection time. In addition, we checked whether STE has a virucidal activity on IAV. FACS analysis and fluorescence microscopy results showed that, in the presence of STE, the IAV attachment was significantly suppressed and that the viral entry was slightly reduced (Figure 4). In addition, STE exerted moderate virucidal activity. These results imply that STE has a substantial inhibitory effect against IAV infection by hindering viral binding to the cells and inducing virucidal action. 

### 3.5. STE Inhibits Hemagglutination

**Figure 5:**
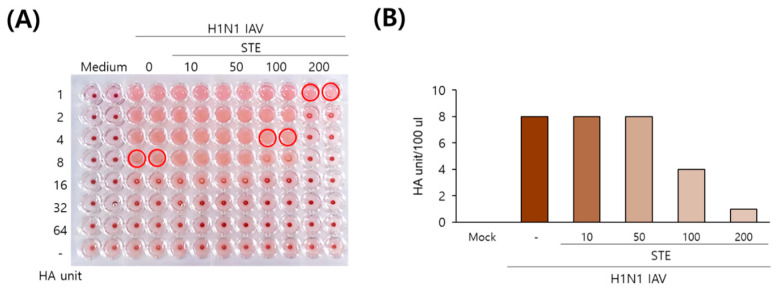
STE reduces the HA unit of influenza viruses (**A**,**B**). The cells were cotreated with STE at the indicated concentrations and H1N1 IAV for 24 h at 37 °C. The 2-fold serially diluted supernatants and chicken RBC cells were mixed in round 96-well plates for 1 h at room temperature. The red circle indicates hemagglutination (HA) units.

Hemagglutination induced by IAV hemagglutinin, which is closely related to viral binding to the cells, is a main target for anti-influenza viral drugs. As we found that STE has an inhibitory effect on IAV binding to the cells, we next explored whether STE could repress IAV hemagglutination on red blood cells (RBCs). As presented in Figure 5, STE dose-dependently decreased the HA units of the H1N1 IAV. The HA units of the control H1N1 IAV were 8 units. However, in the presence of 100 μg/mL or 200 μg/mL of STE, the HA units of the virus were 4 or 2 units, respectively. STE decreased the HA units by 2- or 4-fold compared to the virus-infected control. These results imply that STE exerts an inhibitory effect on hemagglutination at concentrations of 100 or 200 µg/mL.

### 3.6. STE Represses Neuraminidase Activity of H1N1 and H3N2 IAVs

**Figure 6:**
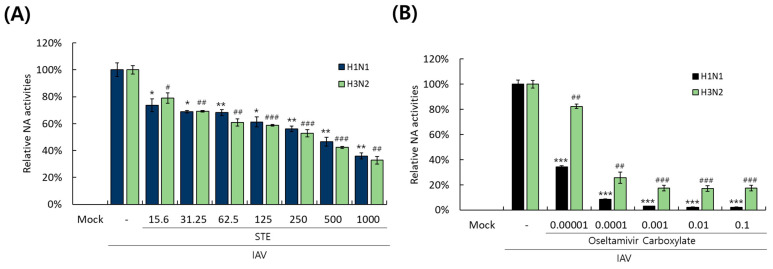
STE dose-dependently represses the neuraminidase activities of H1N1 and H3N2 IAVs. Serially diluted STE (**A**) or oseltamivir carboxylate (**B**) was mixed with H1N1 or H3N2 IAV in 96-well black plates. The detailed neuraminidase activity assay was described in materials and methods. The data represent the mean ± SD from three independent experiments. The unpaired Student *t*-test was used to assess the statistical significance. * *P* < 0.05, ** *P* < 0.005, and *** *P* < 0.0005, compared with the H1N1 virus-infected group. ^#^ *P* < 0.05, ^##^ *P* < 0.005, and ^###^ *P* < 0.0005, compared with the H3N2 virus-infected group.

Since the neuraminidase activity is indispensable for viral progeny release from the cells after replication, we next examined whether STE could inhibit the NA activities of H1N1 and H3N2 influenza viruses. Figure 6A presents that STE dose-dependently reduces the NA activities of H1N1 and H3N2 IAVs. In particular, STE at 500 µg/mL potently inhibited the NA activities of more than 50% of the virus-infected control. Oseltamivir carboxylate, a positive control, exerted a potent inhibitory effect on the NA activities of H1N1 and H3N2, dose-dependently (Figure 6B). 

## 4. Discussion

ST is known as a medicinal herb effective on various ailments. Our research group has demonstrated that ST has a neuroprotective effect and an inhibitory effect on osteoclastogenesis in previous reports. Several research groups have demonstrated that ST has antiviral efficacy against herpes and coxsackie viruses. However, the inhibitory effect of STE against the influenza virus was not revealed until now. In this study, we discovered that STE has an inhibitory impact against influenza virus infection. The CPE inhibition assay showed that STE significantly protected the cells from the H1N1 and H3N2 IAVs, as well as from PR8-GFP IAV infection. The time-of-addition assay showed that the anti-viral effect of STE arises from blocking viral binding to the cells and inducing the direct killing of the virus at an early phase upon infection. When we conducted the hemagglutination inhibition assay to address whether ST could affect HA, which is critical for IAV binding to the cells, we found that STE significantly blocked the HA of IAV. These results imply that STE inhibits the virus binding to the cells by interfering with the HA of IAV. Several reports have shown that the inhibitory effect of herbal extracts, such as *Eupatorium perfoliatum* L., *Paeonia lactiflora*, *Isatidis Radix*, *Alpinia katsumadai*, *Alchemilla mollis*, *Jatropha curcas Linn*. leaf, against NA of IAV, is closely related to viral binding to the cell membrane [20,21,22,23,24,25,26,27]. We also demonstrated that medicinal herbal extracts, including *Thuja orientalis folium* [19] and *Hoveniae Semen Seu Fructus* [28], attenuate IAV infection by modulating HA. STE dose-dependently repressed the neuraminidase activities of H1N1 and H3N2 IAVs. Neuraminidase is the key enzyme for the release of viral progeny in the later stages of IAV infection. Most antiviral drugs for IAV, including oseltamivir or zanamivir, target the NA of IAV. Natural products such as *Geranii* herba [10], *Rhus verniciflua* [29], *Salvia plebeia R. Br*. [30], and chemicals, including chlorogenic acid, catechin, fucoidan, isoimperatorin, and oroxylin A [31,32,33,34,35], were reported to repress the neuraminidase of IAV. STE also exhibited the direct killing effect at the early stage of IAV infection. The virucidal effect has been reported to be related to certain components in the extract that directly bind to viruses and block viral particles from attaching to cells [25,36,37]. In the previous report, amentoflavone was identified as the main compound of ST in HPLC analysis [18]. Additionally, we found that amentoflavone was one of the anti-influenza viral components of *Thuja orientalis folium* [19]. These results suggest that amentoflavone may play a role in anti-influenza viral activity in ST. Bailly C. reported that ST, which contains many phenolic compounds, is used in traditional medicine and in cosmetics to protect the skin and has applications in vegetable dishes and beverages [14]. Although further studies are needed to determine which ingredients in ST are involved in inhibiting influenza virus infection, ST has the potential to be utilized as a material to inhibit the influenza virus. In summary, ST exerts its antiviral activity by inhibiting HA and NA, thereby impairing the attachment and release of influenza viruses.

## 5. Conclusions

STE exerted a potent anti-influenza A viral activity. STE significantly blocked influenza virus infection via the inhibition of hemagglutinin, which is involved in virus attachment. Further, STE inhibited the activity of the IAV neuraminidase. Our results suggest that STE could be used as an anti-influenza viral ingredient in vegetable dishes, beverages, or teas, as well as a natural agent against influenza virus infection.

## Data Availability

Data are contained within the article and Supplementary Materials.

## Supplementary Materials

The following supporting information can be downloaded at: https://www.mdpi.com/article/10.3390/nu16142377/s1. Figure S1: Cytotoxicity of STE on MDCK and A549 cells; Figure S2: The inhibitory effect of STE against PR8-GFP IAV infection in MDCK and A549 cells.
