# Supplementary material for: Selaginella tamariscina Ethanol Extract Attenuates Influenza A Virus Infection by Inhibiting Hemagglutinin and Neuraminidase
Source: Nutrients. 2024 Jul 22;16(14):2377. doi: 10.3390/nu16142377 (PMC11280371; doi:10.3390/nu16142377)
Supplement: Supplementary file 1 [file nutrients-16-02377-s001.zip › nutrients-3089496-supplementary.pdf]

Supplementary data

Figure S1.

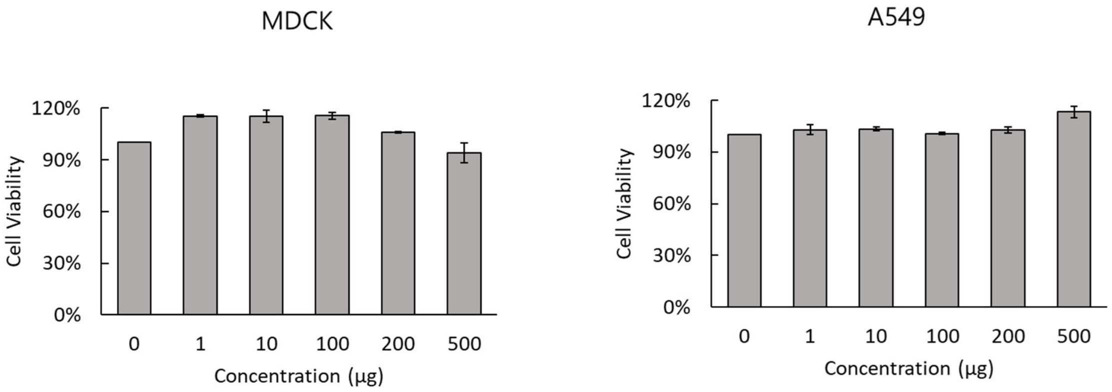

Cytotoxicity of STE on MDCK and A549 cells. STE at the indicated concentrations was treated in the cells for 24 h. The cell viability was determined using CCK-8 assay. The data represent the mean ± SD based on three replicates in three different experiments.

Figure S2.

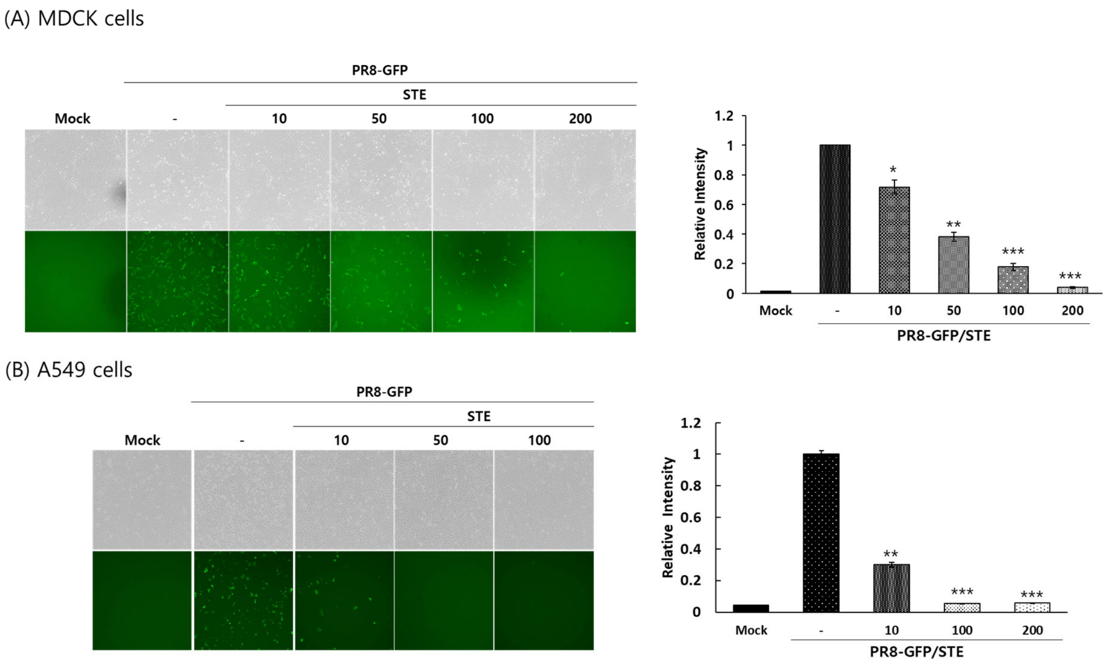

The inhibitory effect of STE against PR8-GFP IAV infection in MDCK and A549 cells. STE at the indicated concentrations or medium (Mock) mixed with PR8-GFP IAV for 1 h at 4°C were cotreated to the cells for 2 h at 37°C. After washing with PBS, the cells were further incubated for 24 h. The levels of GFP expression were evaluated with fluorescent microscopy and FACS analysis.
